# Combination of Continuous Use of Oral Clomiphene Citrate with Injectable Gonadotropins for Ovarian Stimulation: A Single-Center Study

**DOI:** 10.3390/life15081235

**Published:** 2025-08-04

**Authors:** Adamantia Kontogeorgi, Gkalia Tsangkalova, Panagiota Ambatzi, Ioannis Boutas, Eleftherios Meridis, Ioannis Gryparis, Dimitrios Kalaitzis, Angeliki Fenga, Melpomeni Peppa, Sophia Kalantaridou, Antonios Makrigiannakis, Minas Paschopoulos

**Affiliations:** 1 School of Medicine, University of Crete, 710 03 Heraklion, Greece; 2 IVFSerum Advanced IVF Treatment Center, 141 23 Likovrisi, Greece; 3 Rea Maternity Clinic, 175 64 Palaio Faliro, Greece; ioannis.boutas@gmail.com; 4 Independent Authority of Public Revenue, 101 84 Athens, Greece; 5 2nd Department of Internal Medicine, Research Institute and Diabetes Center, National and Kapodistrian University of Athens, Attikon University Hospital, 124 62 Chaidari, Greece; 6 School of Medicine, National and Kapodistrian University of Athens, 106 79 Athens, Greece; 7 School of Medicine, University of Ioannina, 455 00 Bizani, Greece

**Keywords:** clomiphene citrate, embryo banking, ovarian stimulation protocol, frozen embryo transfer, gonadotropins

## Abstract

**Objective**: This retrospective observational study evaluated the efficacy and safety of an ovarian stimulation protocol for embryo banking that involves continuous administration of clomiphene citrate (CC) in combination with gonadotropins, without the use of GnRH antagonists. **Methods**: Conducted at the Serum IVF Clinic in Athens, Greece, the study included 250 women aged 25–45 who underwent IVF for embryo banking. The protocol involved administering 150 mg of CC daily from day 2 of the menstrual cycle until the day before hCG trigger, alongside 150 IU/day of Meriofert. Outcomes assessed included oocyte yield, fertilization rates, incidence of ovarian hyperstimulation syndrome (OHSS), and hormonal correlations. Comparative and regression analyses explored differences between age groups and predictors of success. **Results**: The protocol demonstrated a favorable safety profile with no cases of OHSS and yielded a mean of 10.25 oocytes per patient. Group analysis showed significantly more oocytes retrieved in women under 40 (mean: 12.5) versus those over 40 (mean: 8.43), while fertilization rates were paradoxically higher in the older cohort (59.16% vs. 30.68%, *p* < 0.0001). Regression models revealed basal FSH to be a significant inverse predictor of oocyte yield, but it was positively associated with fertilization rate. Continuous CC use effectively suppressed premature LH surges without compromising oocyte or embryo quality, allowing flexible and cost-effective stimulation with minimal monitoring. **Conclusions**: Continuous administration of clomiphene citrate in combination with gonadotropins presents a promising, antagonist-free ovarian stimulation protocol for embryo banking. The approach is economically efficient, reduces monitoring requirements, and maintains safety and effectiveness and is particularly notable in women over 40. Further studies are warranted to validate these findings and refine protocol mechanisms.

## 1. Introduction

The use of IVF techniques has made significant progress, with many protocols developed to increase the success rates of IVF and improve the overall procedure [1]. The rotations of frozen embryo transfer (FET) are of international importance [2], owing to the continued development of vitrification equipment and innovation [3]. Among the essential factors contributing to the increase in frozen embryo transfer are (i) the use of alternative ovarian stimulation protocols to prevent ovarian hyperstimulation syndrome [4], (ii) the increasing use of pre-implantation genetic testing for aneuploidies (PGT-A) [5], and (iii) the introduction of elective single-embryo transfer policies [6]. Additionally, delaying embryo transfer enables patients to be more flexible in order to plan the procedure in parallel with personal schedules, such as work, family, travel, etc.

Improving ovarian stimulation is essential for embryo banking because it directly influences both the quantity and quality of retrieved oocytes, which in turn affects fertilization rates and embryo viability. Traditionally, a high dose of gonadotropins was used to improve oocyte yield. Although this method is considered outdated in numerous circumstances [7], it has been proven effective for both high and low responders, resulting in the formation of extra embryos and extra embryos available for cryopreservation [8]. The use of gonadotropin-releasing hormone (GnRH) analogues and antagonists prevents early luteinizing hormone (LH) surges and ovulation, allowing the administration of increased gonadotropin doses [9]. While this strategy has a significantly higher success rate in traditional IVF, it also has certain challenges, including higher medication costs and the need to monitor frequently through multiple scans in order to develop a custom ovarian stimulation protocol [10].

To balance productivity, effectiveness, and cost, the development of a reinforced, cost-efficient protocol for FET cycles is essential [10]. Clomiphene citrate (CC), an inhibitor of natural steroid hormones like estradiol, has emerged as a viable option [11]. It has demonstrated an improvement in IVF success rates by using lower doses of injectable gonadotropins, making it a cost-effective and competitive solution [12]. There are several studies proposing numerous ovarian stimulation regimens combining CC with gonadotropins, recombinant follicle-stimulating hormone (FSH), and luteinizing hormone (LH). Protocols involving this combination have shown promising results, particularly in the quantity and quality of retrieved oocytes [13]. Nevertheless, the success of CC-based protocols means that they are largely restricted to 5 days of usage at the start of the cycle. Research has shown that continuous use of clomiphene can suppress endogenous LH surges at the hypothalamic level, a finding that has yet to be fully integrated into existing stimulation protocols [14].

The present observational study delves into the research of a new technique involving continuous administration of gonadotropins and clomiphene citrate from the beginning of the cycle until the trigger shot with human chorionic gonadotropin (hCG), under the context of embryo banking cycles without the use of the GnRH antagonist. The objective of the study is to evaluate the effects and outcomes of the present protocol and to provide an understanding of the advantages of its efficiency and capability.

## 2. Materials and Methods

This study was a single-center, retrospective observational study conducted to assess the outcomes of an ovarian stimulation protocol for embryo banking. The study was performed at Serum IVF Clinic in Athens, Greece, a fertility clinic specializing in assisted reproductive technology since 1989. The protocol applied continuous use of clomiphene citrate combined with gonadotropins during ovarian stimulation cycles. Ethical approval was obtained from the institutional review board (Application protocol number: 13054/13 May 2025, Board decision protocol number: 13055/14 May 2025), and all patient data were anonymized to maintain confidentiality. This initial study was designed as a retrospective observational analysis with the primary aim of reporting the outcomes and safety data of an ovarian stimulation protocol. At this stage, the goal was not to directly compare this approach with existing protocols, but rather to establish a foundational understanding of its clinical performance and to assess its feasibility, safety, and clinical results. Emphasis was placed on characterizing how the protocol functions as a standalone strategy, particularly in the context of embryo banking. Women undergoing ovarian stimulation cycles for embryo banking during the study period were included. Eligible participants met the following inclusion criteria: Age between 25 and 45 years, undergoing ovarian stimulation for embryo banking, and using the Clomid-based stimulation protocol as described in this study. Exclusion criteria included women with contraindications to clomiphene citrate, ovarian stimulation cycles conducted for immediate fresh embryo transfer, and incomplete medical records.

To enhance the consistency of the study sample, all participants had no medical history of male factor infertility. Semen analysis confirmed that all sperm samples had a DNA fragmentation index (DFI) below 15%, a concentration exceeding 15 million/mL, motility above 40%, and morphology greater than 4%. To exclude uterine factor infertility, all female participants underwent a pelvic scan, an aqua scan, and microbiome testing, which confirmed normal uterine anatomy and a healthy endometrial microenvironment. Additionally, women with an endometrial thickness below 9 mm at the time of embryo transfer were excluded from the study. This rigorous selection process aimed to ensure a homogeneous sample, allowing an accurate evaluation of the stimulation protocol’s efficacy.

**Figure 1:**
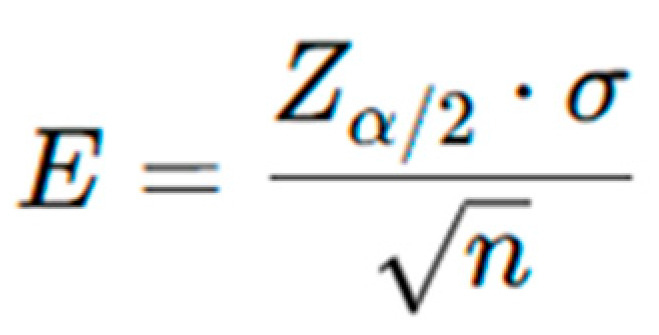
The margin of error (E) in confidence intervals equation.

**Figure 2:**
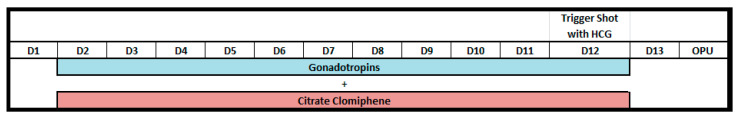
Schematic illustration of the continuous CC administration protocol. D—Day of menstrual cycle.

A power analysis determined that a sample of 96 participants would provide a 95% confidence level with an error margin of 0.2. However, the available sample size was increased to 250 participants, thereby reducing the margin of error below 0.1 and improving the precision and reliability of the estimates. As shown in Figure 1, the margin of error (E) decreases with the square root of the sample size (n), enhancing confidence in the study’s conclusions. The ovarian stimulation protocol included continuous use of clomiphene citrate (150 mg daily from day 2 of the cycle) with gonadotropins (Meriofert, 150 IU/day), a combination of FSH and LH. The stimulation lasted 10–12 days without antagonist administration. Final oocyte maturation was triggered with hCG as clinically indicated, followed by ultrasound-guided oocyte retrieval 36 h later (Figure 2).

The primary outcomes were oocyte retrieval rate, embryo development, and clinical pregnancy rates post-embryo transfer. The secondary outcomes included total gonadotropin dose and incidence of OHSS. The data were analyzed using the SPSS software (version 29). Continuous variables were reported as mean standard deviation (SD) or median with interquartile range (IQR), depending on distribution; categorical variables were reported as frequencies and percentages. Ethical approval was obtained from the Institutional Review Board and the National Committee of Reproductive Medicine (13055/14 May 2025). Informed consent was waived due to the retrospective nature of the study, and all data were anonymized.

## 3. Results

### 3.1. Descriptive Analysis

A total of 250 IVF patients were included in the study, with a mean age of 39.10 ± 4.33 years. Demographic characteristics are summarized in Table 1, including body mass index (BMI) at 23.35 ± 2.25 kg/m^2^, antral follicle count (AFC) at 9, and infertility duration averaging at 2 years. The baseline hormonal levels were as follows: Basal-E2 at 128.73 pmol/L, Basal-PRG at 1.08 nmol/L, Basal-LH at 5.54 mIU/mL, and Basal-FSH at 7.57 mIU/mL. Notably, the cohort exhibited a wide range of AMH values (0.10–7.29 ng/mL) with the mean being 4.29 ng/mL. All the participants underwent ovarian stimulation using a continuous Clomid protocol combined with injectable gonadotropins, without GnRH antagonist administration, relying instead on the endogenous suppression of LH by Clomid.

The cycle characteristics are also detailed in Table 1. Each patient received an initial dose of 150 IU of urofollitropin (FSH + LH), with a mean total gonadotropin dose of 1502.41 ± 98.23 IU and a median stimulation duration of 10.07 days (range: 8–12). Fertilization was performed using either IVF or ICSI, achieving a rate of 46.41% when including all the oocytes retrieved, regardless of the maturity or morphological status. When considering only mature oocytes, the fertilization rates were 78% with ICSI and 81% with IVF. All the embryos were cultured for five to six days, at 37 °C, 6.0% CO_2_, and cryopreserved at the blastocyst stage. The culture media were the sequential (G1 PLUS, G2 PLUS) of the G series (Vitrolife, Gothenburg, Sweden). Embryos were cryopreserved at the blastocyst stage using the vitrification method.

The blastocyst formation rate was approximately 32%, indicating a consistent development of embryos to the blastocyst stage under the applied culture conditions. Post-thaw survival rate following vitrification was remarkably high, reaching 99%, confirming the efficiency and reliability of the cryopreservation protocol used. All day-5 and day-6 blastocysts were evaluated using Gardner’s grading system, which assesses blastocyst expansion, inner cell mass (ICM), and trophectoderm (TE) quality. Only blastocysts meeting the morphological criteria for transfer or cryopreservation were included in further procedures. The pregnancy rate was 29.75% in women under 40 years of age and 19.97% in those aged 40 and above. Reliable data on live birth rates are not available, as patient follow-up is conducted only through the end of the first trimester, after which care is transferred to obstetric providers.

Importantly, no cases of ovarian hyperstimulation syndrome (OHSS) were observed, regardless of oocyte yield or estradiol levels on trigger day, suggesting that the Clomid-based protocol offers both efficacy and safety, even in high responders.

### 3.2. Group Analysis and Comparison

To better understand the result based on the individual characteristics of the patients in the sample, we divided the sample into two groups based on their age. The first group was the group for women younger than 40 years of age (Group A), and the second group was for women older than 40 years of age (Group B). A comparative analysis was conducted to evaluate any differences in the number of oocytes retrieved and fertilization rates between the two groups (Table 2).

The mean number of oocytes retrieved was higher in Group A (M = 12.50) compared to Group B (M = 8.43). Statistical analysis using an independent *t*-test revealed a significant difference between the groups. Under the assumption of equal variances (Student’s *t*-test), the difference was statistically significant (t = 5.18, *p* < 0.0001). A separate analysis with unequal variance (Welch’s *t*-test) also confirmed significance (t = 4.90, *p* = 2.27 × 10^−6^).

The fertilization rate was higher in Group B (M = 82.4%) compared to Group A (M 80.0%). This difference was statistically significant under both equal and unequal variance assumptions. The equal variance *t*-test yielded a significant result (t = −2.12, *p* = 0.035), while the unequal variance *t*-test confirmed the significance of the difference (t = −2.14, *p* = 0.034). Although the difference is modest, it is statistically meaningful and suggests that, contrary to expectations, women over 40 in this cohort demonstrated slightly higher fertilization rates than their younger counterparts (Table 3).

The analysis demonstrated that younger patients had a significantly higher number of retrieved oocytes, as was anticipated, while older patients exhibited a higher fertilization rate. These findings suggest that while ovarian reserve declines with age, embryo quality or other factors may contribute to improved fertilization outcomes in older patients. The embryo quality assessment occurred on the blastocysts, on their 5th and 6th day, according to Gardner’s grading system.

### 3.3. Analytical Statistics Regarding Cycle Parameters

Multiple regression analyses were conducted separately for women aged 40 and under and those over 40, to evaluate potential predictors of fertilization rate, including age, AMH, AFC, and the number of oocytes retrieved. In the younger cohort, the model yielded an R^2^ value of 0.015, indicating that only 1.5% of the variability in fertilization rate could be explained by the selected predictors. Similarly, in the older cohort, the R^2^ was 0.019, with 1.9% of variability explained. In both groups, none of the predictors demonstrated statistical significance, suggesting that other unmeasured biological or laboratory factors may be more influential in determining fertilization outcomes.

Importantly, all patients underwent a uniform ovarian stimulation protocol and intracytoplasmic sperm injection (ICSI), ensuring consistency in clinical and embryological procedures. Interestingly, the average fertilization rate was marginally higher in the older age group (82.4%) compared to the younger group (80.0%), a counterintuitive finding given the expected decline in oocyte quality with age. This may reflect subtle biological differences in oocyte maturation, laboratory handling, or responsiveness to stimulation protocols that are not adequately captured by traditional ovarian reserve parameters.

### 3.4. Suggested Interpretation of the Mechanism of Protocol Action

In addition to the clinical effectiveness of the protocol, the observations concerning the correlations in the mechanism of action of the protocol are also important. Initially, we observed that at older ages there are better fertilization rates, as recorded in our sample. This was followed and backed up by a correlation study between the values of FSH and LH before stimulation and their impact on the fertilization rate. Multiple linear regression models were constructed to evaluate the association of basal LH and FSH levels with the number of oocytes retrieved and the fertilization rate.

For the outcome of oocytes retrieved, the model, which included basal LH and FSH as predictors, explained 9.3% of the variance (R^2^ = 0.093). In this model, basal FSH was significantly and inversely associated with the number of oocytes retrieved (β = –0.71, *p* = 0.00091), indicating that higher FSH levels were related to a lower oocyte yield. In contrast, basal LH did not show a significant association (β = –0.03, *p* = 0.91).

A separate regression analysis was performed with the fertilization rate as the dependent variable. The overall model accounted for 9.8% of the variation in fertilization rate (R^2^ = 0.098). Here, basal FSH was positively associated with fertilization rate (*β* = 2.09, *p* = 0.0075), suggesting that despite the reduced number of oocytes retrieved with higher FSH, the fertilization rate among the retrieved oocytes was increased. Basal LH was not significantly associated with fertilization rate (*β* = 1.01, *p* = 0.29).

These findings indicate that while basal FSH is a significant predictor in both models and while it negatively influences oocyte yield and positively influences fertilization rate, basal LH does not appear to contribute significantly to these outcomes in our sample.

Lastly, the presented protocol refers to ovarian stimulation that does not require the administration of an antagonist. Clomiphene is known to have estrogenic and anti-estrogenic effects; so its administration continuously in a cycle can replace the use of an antagonist [32]. A linear regression analysis was performed to examine the relationship between basal estradiol (E2) levels before stimulation and progesterone (PRG) levels on the day of the trigger. The model demonstrated a statistically significant association (*p* = 0.00023); however, the relationship was weak, with an R^2^ value of 0.054, indicating that only 5.4% of the variation in PRG levels could be explained by basal E2 levels.

The regression coefficient (*β* = 0.0017) suggests a minor positive correlation, where higher basal E2 levels before stimulation were associated with slightly higher PRG levels on the trigger day. Despite reaching statistical significance, the low R^2^ value highlights that estradiol before stimulation is not a strong predictor of progesterone at the trigger, suggesting that other hormonal or ovarian factors contribute more significantly to PRG regulation during stimulation cycles.

We believe that further research, taking into consideration additional endocrine markers, ovarian response parameters, and individualized stimulation protocols, is required to better understand the hormonal interplay influencing PRG levels at the time of the trigger. Relevant literature on the continuous administration of clomiphene citrate in ovarian stimulation protocols was reviewed to contextualize and compare the outcomes of our study with those reported by other researchers, as can be seen in Table 3. 

## 4. Discussion

### 4.1. Clomiphene Mechanism of Action

Clomiphene citrate (CC) has been a cornerstone of fertility treatments for over four decades, essentially employed due to its cost-effectiveness and efficiency in inducing ovulation. Its mechanism of action includes its functioning as a selective estrogen receptor modulator (SERM) and the role it plays as a competitive antagonist to estrogen at receptor binding sites in the central nervous system [33]. The primary site of clomiphene citrate’s action is the hypothalamus, where it blocks estrogen receptors and thus disrupts the negative feedback loop of circulating endogenous estrogen [34]. This leads to increased levels of gonadotropins, particularly luteinizing hormone (LH) and follicle-stimulating hormone (FSH), in the bloodstream [35].

Historically and over time, the standard technique has been to administer CC during the early follicular phase, usually occurring on the second or third day of the cycle and persisting for a total of 5 days. In order to reduce the required dosage of gonadotropins and, accordingly, the cost of the treatment, the procedure is commonly followed by an overlap of gonadotropin stimulation. Furthermore, gonadotropins suppress CC’s negative effects on the endometrium [32]. However, a major drawback of this method is the frequent occurrence of premature LH surges, which leads to premature luteinization prior to the follicles achieving full maturation, which frequently leads to high cycle cancellation rates. Research by Al-Inany et al. [36] demonstrated the benefits of using CC continuously in ovarian stimulation protocols with gonadotropins for intrauterine insemination (IUI), especially in preventing premature LH surges. Their assumption was that CC’s anti-estrogenic properties suppress premature LH increases while still promoting ovarian follicle growth. In another study, a combination of gonadotropins and CC during the mid-to-late follicular phase was compared with a short protocol using gonadotropin-releasing hormone (GnRH) agonists and gonadotropins. The two protocols have comparable pregnancy outcomes, with the gonadotropin–CC protocol having the extra advantage of avoiding premature LH surges.

More recently, Shams-Eldeen et al. [37] performed a pilot study evaluating the possibility of extending the use of CC beyond the standard 5-day regimen in combination with gonadotropins. Their findings suggest that prolonging CC administration effectively prevents premature LH surges while retaining oocyte output, highlighting its potential as an alternative protocol. CC suppresses the LH surge by acting as a SERM that disrupts the normal feedback mechanisms of the hypothalamic–pituitary–gonadal (HPG) axis. At the molecular level, CC binds competitively to estrogen receptors, particularly ER-alpha, in the hypothalamus. This binding prevents estradiol, the primary endogenous estrogen, from exerting its effects on the hypothalamus. Normally, estradiol has a negative impact on the hypothalamus, reducing the secretion of gonadotropin-releasing hormone (GnRH) at a time when sufficient follicular development has taken place. By blocking this feedback loop, CC suppresses the hypothalamus’s ability to sense and respond to estrogen levels [38].

With feedback inhibition suppressed, the hypothalamus increases the frequency and amplitude of GnRH pulses, which, in turn, stimulates the anterior pituitary to release increased amounts of FSH and LH. During early follicular development, this effect supports the recruitment and growth of ovarian follicles. However, when CC is used continuously or beyond the typical five-day regimen, its anti-estrogenic effects extend beyond follicular recruitment. CC reduces the hypothalamus’s sensitivity to estradiol’s positive feedback, which is critical for triggering the pre-ovulatory LH surge. Even as estradiol levels rise due to growing follicles, the pituitary gland does not receive sufficient stimulatory feedback to initiate a premature LH surge. This suppression of positive feedback is central to CC’s ability to prevent early luteinization and premature ovulation. Furthermore, CC interferes with estrogen receptor signaling in kisspeptin neurons, located in the hypothalamic arcuate nucleus, which play a crucial role in regulating GnRH secretion [39]. This disruption reduces kisspeptin-mediated activation of GnRH neurons, further suppressing the hypothalamic–pituitary axis and ensuring that the LH surge does not occur prematurely. Additionally, CC exerts peripheral anti-estrogenic effects at the level of the pituitary. By blocking estrogen’s positive feedback on the pituitary, CC maintains steady gonadotropin secretion while preventing the typical LH surge that accompanies ovulation.

As for the effect of clomiphene citrate (CC) on the endometrium, it is well established that continuous administration from the early follicular phase is associated with endometrial thinning, primarily due to CC’s prolonged anti-estrogenic action. As a selective estrogen receptor modulator (SERM), clomiphene competitively binds to estrogen receptors, stimulating gonadotropin release via the hypothalamic–pituitary axis while exerting a peripheral antagonistic effect on estrogen-responsive tissues such as the endometrium. This sustained receptor blockade disrupts estrogen-driven endometrial proliferation, resulting in impaired stromal and glandular development and leading to suboptimal endometrial thickness. The adverse impact becomes more pronounced with extended CC exposure, which often necessitates the deferral of embryo transfer and favors frozen embryo transfer (FET) in subsequent hormonally regulated cycles to ensure optimal endometrial receptivity. In our study, the mean endometrial thickness measured was 4 mm, a value considered inadequate for fresh embryo transfer [40].

### 4.2. Current Literature for Continuous Clomiphene Administration in Combination with Injectable Gonadotrophins

Regardless of its suppressive effects on the LH surge, CC allows continued FSH production that promotes follicular growth and maturation. This controlled hormonal environment ensures that each follicle develops to an optimal size and maturity without the risk of premature ovulation or luteinization. These mechanisms together explain how CC regulates the hormonal axis in order to suppress the LH surge and provide a solid platform for controlled ovarian stimulation and oocyte retrieval. CC disrupts the molecular pathways that would otherwise have triggered premature ovulation by interfering with estradiol signaling in both the hypothalamus and the pituitary.

Despite its effectiveness as a continuously administered ovarian stimulator, few studies have explored this present implementation in detail. Only 19 studies were carried out between 1988 and 2020, analyzing ovarian stimulation using CC in combination with gonadotropins. Most protocols utilized CC at doses of 50–150 mg per day, administered during the early follicular phase, starting on the second or third day of the menstrual cycle and continuing for 5–6 days. HMG doses varied significantly, with some studies using relatively low doses of 75–150 IU per day [15,16], while others employed higher doses of 300–450 IU per day [13,17], likely tailored to specific patient populations. GnRH antagonists were included in most of these protocols in order to prevent premature LH surges, but some studies, such as those performed by Ashrafi et al. [18] and Ochin et al. [19], omitted GnRH antagonists, relying instead on endogenous suppression of LH.

In particular, only three studies have investigated extended CC administration beyond the usual 5-day period. For instance, Ochin et al. [19] and Singh et al. [12] extend CC use up to one day before the trigger shot, using CC’s suppressive effects on premature LH surges without any compromise in oocyte production. This prolonged use of CC is perfectly consistent with the protocol presented in this article, which involves continuous CC administration from day 2 of the cycle until one day before the trigger shot, without GnRH antagonist use. The details of these three studies can be seen in Table 4. The main objective of the present protocol is to provide an economically competitive option for embryo banking cycles, which provides flexibility in ultrasound monitoring of ovarian stimulation. This procedure additionally reduces costs by integrating the first ovarian ultrasound on the eighth day of the cycle and eliminating the need for antagonist initiation. The suppression of the LH surge reduces the risk of ovulation and thus reduces the need for close monitoring.

All the studies share the compelling observation that continuous clomiphene citrate use appears to reduce the incidence of OHSS [41]. A plausible mechanism involves the blockade of estrogen receptors at the hypothalamic level, leading to increased endogenous FSH and LH secretion. Moreover, clomiphene is known to exert a ceiling effect, limiting excessive multifollicular development, even at higher doses. Nonetheless, further prospective studies are needed to validate these preliminary findings and explore additional mechanistic insights.

### 4.3. New Insights

An important insight arising from our findings is the potential clinical value of antagonist-free stimulation protocols not only in terms of safety, but also in supporting individualized treatment approaches. The combination of clomiphene citrate and gonadotropins, without GnRH antagonists, demonstrated effective follicular recruitment and fertilization outcomes, with a notably low risk of ovarian hyperstimulation even among high responders. This observation suggests that such protocols could be particularly advantageous in patients at risk for OHSS or in resource-limited settings where cost containment is essential. Moreover, the consistently high post-thaw survival rate of blastocysts supports the use of deferred frozen embryo transfer as a viable strategy to bypass the negative endometrial effects of clomiphene while preserving embryo viability.

Interestingly, statistical analysis revealed that despite producing fewer oocytes on average, women in the older age subgroup achieved comparable—if not better—fertilization and blastocyst development rates than their younger counterparts. This finding suggests that the protocol may favor oocyte quality over quantity in older women, potentially due to a more physiologic, less aggressive stimulation environment. The controlled endocrine milieu created by clomiphene citrate and moderate gonadotropin dosing might support more synchronized follicular growth and better cytoplasmic maturation of oocytes in this age group. These results challenge the traditional assumption that older women universally require high-dose stimulation and instead highlight the importance of individualized, physiology-respecting approaches in advanced maternal age. Further prospective studies are needed to validate whether such protocols can routinely enhance clinical outcomes for women over 38 years old. These results prompt further exploration into how simplified stimulation protocols can be safely and effectively adapted across diverse patient populations, particularly when paired with vitrification and precise endometrial preparation in subsequent cycles.

## 5. Conclusions

Without a doubt, IVF is the preferred choice for many women today, and the scientific community unquestionably must provide options and personalized stimulation protocols. Embryo banking cycles, in combination with a continuous clomiphene administration protocol, offer, according to the observations conducted and presented in this article, flexibility and effectiveness, adequately addressing the needs of many patients. The authors recognized that, in order to design and accurately assess the efficacy of an ovarian stimulation protocol, it is essential to eliminate confounding factors related to male infertility and uterine conditions. However, we acknowledge that the sample size in this study may not provide sufficient statistical power to comprehensively address all of these variables. This limitation could potentially be overcome through future studies with larger sample sizes or by combining data from multiple similar studies, thereby strengthening the overall statistical significance. There is no doubt that additional similar studies utilizing comparable stimulation regimens are necessary so that the scientific community can establish a definitive stance on the efficacy of this specific protocol. However, it is evident that the continuous action of clomiphene allows ovarian stimulation with lower doses of gonadotropins, without the need for an antagonist, at a lower cost and with reduced ultrasound monitoring. The results appear promising in terms of fertilization rates in women over 40 years old, while avoiding the risk of ovarian hyperstimulation syndrome (OHSS). Further studies may help clarify the mechanism of action of this treatment regimen and extend its application to other patient groups.

## Figures and Tables

**Table 1:** Demographic and cycle parameter characteristics of the participants. BMI—Body Mass Index; AFC—Antral Follicle Count; E2—Estradiol; PRG—Progesterone; FSH—Follicle-stimulating Hormone; AMH—Anti-Mullerian Hormone; IVF—In Vitro Fertilization.

**Demographic Characteristics** **(Total Sample *n* = 250)**	**IVF Patients (*n* = 250)**	**40 and Below (*n* = 113)**	**41 and Above (*n* = 137)**
Age	39.10 ± 4.33	35.9	42.65
BMI (kg/m^2^)	23.35 ± 2.25	23.25	23.46
AFC	9 (35-1)	12	6
Infertility duration (years)	2 (6-1)	2.5	2.3
Basal-E2 (pmol/L)	128.73 (368.00-23.00)	149.16	105.48
Basal-PRG (nmol/L)	1.08 (1.70-0.40)	1.1	1
Basal-LH (mlU/mL)	5.54 (14.00-2.40)	5.26	8.64
Basal-FSH (mlU/mL)	7.57 (16.20-3.80)	7	9.21
AMH (ng/mL)	4.29 (72.9-0.10)	5.25	1.19
**Cycle Parameters Characteristics**	**IVF Patients (*n* = 250)**	**40 and Below (*n* = 113)**	**41 and Above (*n* = 137)**
Total doses of gonadotropins	1502.41 ± 98.23	1517	1485
Days of stimulation	10.01 (12-8)	10.11	9.9
E2 on trigger day (pmol/L)	2062.24 (11322-230)	2580.83	1472.12
PRG on trigger day (nmol/L)	0.67 (0.76-0.10)	0.78	0.55
No. oocytes retrieved	10.25 (35-1)	11	8.3
No. mature oocytes retrieved	6.25 (14-1)	8.35	4.19
No. embryos created	4.90 (19-0)	6.25	3.55
No. top quality embryos	3.80 ± 3.98 (21-0)	4.1	3.7
Cases of ovarian hyperstimulation syndrome	0	0	0

**Table 2:** Fertilization rate and mature oocytes retrieved for the two separate groups.

	Group A Under 40 (Mean)	Group B Over 40 (Mean)	*t*-Value	*p*-Value	Interpretation
Fertilization rate (%)	30.68	59.16	−11.06	<0.0001	Fertilization rate is significantly higher in patients over 40 (*p* < 0.001)
No. mature oocytes retrieved	12.5	8.43	4.9	<0.0001	Patients under 40 retrieve significantly more oocytes than those over 40 (*p* < 0.001)

**Table 3:** Current literature for continuous CC action in ovarian stimulation. CC—Clomiphene Citrate, HMG—Human Menopausal Gonadotropin, IU—International Units, GnRH-ant—GnRH Antagonist.

Study	Year	Protocol
Singh et al. [12]	2016	CC 50 mg/day AND HMG 150 IU/day AND no GnRH antagonist
Triantafyllidou et al. [13]	2020	CC 100 mg/day, days 3–7 AND HMG 300 IU/day AND GnRH-ant
Jutras et al. [15]	1991	CC 50 mg/day, days 2–6 AND HMG 150 IU/day AND GnRH-ant
Karimzadeh et al. [16]	2010	CC 100 mg/day, days 2–6 AND HMG 150 IU/day AND GnRH-ant
Schimberni et al. [17]	2016	CC 100 mg/day, days 2–6 AND HMG 450 IU/day AND GnRH-ant
Ashrafi et al. [18]	2005	CC 100 mg/day, days 3–7 AND HMG 150 IU/day NO GnRH-ant
Ochin et al. [19]	2018	CC 50 mg/day, day 3—one day before trigger AND HMG 75–150 IU/day NO GnRH-ant
Lin et al. [20]	2022	CC 50 mg/day AND Letrozole 2.5 mg, day 3—one day before trigger AND HMG 75–150 IU/day AND GnRH-ant
Pilehvari et al. [21]	2016	CC 100 mg/day, days 2–6 AND HMG 150 IU/day AND GnRH-ant
Ragni et al. [22]	2012	CC 150 mg/day, days 2–6 AND HMG 150 IU/day AND GnRH-ant
Revelli et al. [23]	2014	CC 100 mg/day, days 2–6 AND HMG 150 IU/day AND GnRH-ant
Fenichel et al. [24]	1988	CC 100 mg/day, days 3–7 AND HMG 300 IU/day AND GnRH-ant
Grochowski et al. [25]	1999	CC 100 mg/day, days 2–6 AND HMG 150 IU/day AND GnRH-ant
Harrison et al. [26]	1994	CC 100 mg/day, days 2–6 AND HMG 150 IU/day AND GnRH-ant
Lin et al. [27]	2006	CC 100 mg/day, days 3–7 AND HMG 300 IU/day AND GnRH-ant
Long et al. [28]	1995	CC 50 mg/day, days 2–6 AND HMG 150 IU/day AND GnRH-ant
Tummon et al. [29]	1992	CC 100 mg/day, days 2–6 AND HMG 150 IU/day AND GnRH-ant
Weigert et al. [30]	2002	CC 50 mg/day AND HMG 150 IU/day AND GnRH-ant
Bermejo et al. [31]	2014	CC 100 mg/day, day 3 AND HMG 150 IU/day AND GnRH-ant

**Table 4:** Characteristics of similar studies compared to suggested protocol.

Study	Clomiphene Citrate Dose	Trigger	Purpose	Age Group (years)
Ochin et al. 2018 [19]	50 mg/day	N/A	Frozen Cycles	22–38
Singh et al. 2016 [12]	50 mg/day	GnRH agonist (leuprolide acetate)	Oocyte Donor Cycles	20–35
Suggested Protocol (CC 150 mg + Gonadotropins + trigger shot with hCG)	150 mg/day (higher dose, stronger anti-estrogenic effect)	hcG (250 mcg = ~6500 IU)	Frozen Cycles	35–45

## Data Availability

The original contributions presented in the study are included in the article, further inquiries can be directed to the corresponding author.

## Abbreviations

The following abbreviations are used in this manuscript:

CC	Clomiphene citrate
OHSS	Ovarian hyperstimulation syndrome
FSH	Follicle stimulating hormone
LH	Luteinizing hormone
FET	Frozen embryo transfer
GnRH	Gonadotropin-releasing hormone
hCG	Human chorionic gonadotropin
IVF	In vitro fertilization
SD	Standard deviation
IQR	Interquartile range
BMI	Body mass index
AFC	Antral follicle count
E2	Estradiol
AMH	Anti-mullerian hormone
PRG	Progesterone
ICSI	Intracytoplasmic sperm injection
SERM	Selective estrogen receptor modulator
IUI	Intrauterine insemination
HMG	Human menopausal gonadotropin
IU	International units
